# Stakeholder Perceptions of Cultural Tourism Transformation in a Karen Hill Tribe Village, Sangkhlaburi, Thailand

**DOI:** 10.12688/f1000research.186965.2

**Published:** 2026-09-16

**Authors:** Noppadol Dharawanij, Angkana Yaiyong

**Affiliations:** 1 Rangsit University, Lak Hok, Pathum Thani, Thailand; 2 Kasetsart University Faculty of Humanities, Bangkok, Thailand

**Keywords:** borderland tourism, Karen culture, cultural heritage tourism, community participation, sustainable tourism, Sangkhlaburi, Thailand

## Abstract

Ethnic diversity, cultural exchange, and multi-layered governance profoundly shape tourism development in borderland regions. The Karen, Mon, Burmese, and Thai communities of Sangkhlaburi District along the Thai Myanmar border have long relied on their rich cultural heritage as the foundation of their tourism potential. This study examines how cultural tourism in a Karen hill tribe village has evolved within the contemporary tourism environment, particularly during the post-COVID-19 era. Using a qualitative interpretive paradigm, data were collected through semi-structured interviews with 23 purposively selected stakeholders comprising government officials, regional authorities, and ethnic community leaders, supplemented by field observations and document analysis. Thematic analysis identified five key dimensions of tourism transformation: (1) cultural and spiritual foundations of tourism appeal; (2) institutional fragmentation and limited community participation in planning; (3) infrastructure and accessibility constraints; (4) deficiencies in destination marketing; and (5) emerging potential for community-based tourism (CBT) and sustainable practices. The findings indicate that meaningful tourism transformation in borderland settings demands inclusive governance, community capacity building, and participatory planning frameworks that safeguard cultural identity while fostering sustainable economic opportunities.

## 1. Introduction

According to the UNWTO (2023), tourism is a key sector of the economy, a staple of regional development and socio-economic transformation, and plays an important role in border and remote areas. Cross-border areas are usually represented by cultural contact as an environment of cultural multiplicities and ethnic mobility, as well as the proximity of geopolitics, which forms unique tourist environments ( Timothy, 2001). The border areas of Thailand have become increasingly important in the Southeast Asian context as more appealing tourist destinations, which can shift the point of tourist arrivals and initiate inclusive development. This is witnessed in the Sangkhlaburi district, located at the border between Thailand and Myanmar, where the Karen, Mon, and Burmese ethnic groups are found in a single area. The Karen hill tribe has rich and dynamic traditions and religious beliefs, as well as unique knowledge systems, which significantly improve its cultural tourism value. Although Sangkhlaburi has rich tangible and intangible heritage, the development of tourism has not been even, being limited by weak infrastructure, weak institutional frameworks, and limited involvement of the local communities in the planning of their tourism ( King & Parnwell, 2011). COVID-19 also disrupted existing tourism systems, further catalyzing the broader turn to sustainability, local tourism experiences, and community-based approaches ( Gössling, Scott, & Hall 2020; McKinsey & Company 2021). During post-pandemic recovery, Sangkhlaburi has to confront not only challenges but also opportunities to redefine its destinations’ tourism model in a way that involves maintaining the cultural identity and improving the resilience of the Sangkhlaburi community ( Butler, 1980).

This study aims to analyze transformations in cultural tourism in a Karen hill tribe village in terms of stakeholders’ roles and interactions, as well as the level of involvement of the local community and the governance system. It examines stakeholder accounts of participation, cultural identity, and governance in a multiethnic borderland setting. This study is placed in the larger context of the post-pandemic world, contributing to the debate on the sustainable and inclusive development of tourism and analyzing the potential role of culturally sensitive and participatory approaches in promoting the sustainability of tourism in peripheral and indigenous destinations ( UNWTO, 2018).

The contribution is a contextual synthesis of five interconnected dimensions, showing how cultural appeal coexists with perceived limits on participation, coordination, access, and marketing. Unlike route-design research, this study examines stakeholder perspectives; it does not develop or validate itineraries.

## 2. Literature review

The theoretical underpinning of this research is an interdisciplinary body of knowledge comprising borderland tourism, cultural heritage, authenticity, governance, and community-based development. Timothy (2001) has shown that political boundaries at the same time are barriers to tourism movement and providers of possible mobility, resulting in dynamic zones where geopolitical and cultural processes overlap. Based on this view, van Houtum and Strüver (2002) redefined borders as socially constructed places that generate hybrid cultural identities: ‘contact zones’ where changes in social and cultural identity are continually negotiated. The lessons learned help improve the understanding of the complexities of the tourism development process in a multiethnic borderland area like Sangkhlaburi. In the academic field of tourism, the issue of cultural authenticity has been placed at the center. Cohen (1988) turned the traditional sense of authenticity on its head by proposing a new one—emergent authenticity—that emerged as a theory of authenticity that has proven very useful for understanding how such identity is socially constructed and negotiated rather than consolidated and established. This is further expanded by Wang (1999), who suggested the term constructive authenticity, in which cultural tourism is seen as a tool to reinforce identity and intercultural and inter-generational/cultural exchange when communities are actively involved in building their own stories. Richards (2011) also argued that cultural tourism will not be sustainable unless communities have control over the image and the interpretation of their heritage. Timothy and Boyd (2003), Ashworth and Tunbridge (1990) and Echtner and Ritchie (1993) have all studied on heritage tourism as a resource and a management problem, whereas García-Hernández et al. (2017) pointed out its dangers when it is an issue of too many tourists at cultural heritage sites. The concept of community-based tourism (CBT) has garnered much academic interest as a way to apply local empowerment, cultural protection, and economic profit to develop local communities. Murphy (1985) advocated a community approach to tourism development that integrates local interests into planning.

CBT can support livelihood improvement and cultural conservation, although success is not assured ( Goodwin and Santilli, 2009). Okazaki (2008) developed a CBT model that focuses on participative processes and equitable benefits. Kontogeorgopoulos (2021) found local leadership, institutional support from the outside, and the existence of favourable enabling conditions to be the key factors for a successful CBT in Thailand. The governance of sustainable tourism has been studied by Bramwell and Lane (2011), Jamal and Getz (1995) and Arnstein (1969) whose citizens participation ladder serves as a model to measure the level of civic involvement. This study was conducted under the policy background of the BCG Economy Model ( Ministry of Tourism and Sports, 2022) in Thailand, as well as the COVID-19 pandemic and recovery plans ( Gössling et al., 2020; UNWTO, 2022; McKinsey & Company, 2021). Authentic representation of local culture vs. tourism commodification served as the core facets of the theme, captured by four works cited, all by authors working at the interface of communication and tourism: destination marketing and digital communication ( Hays et al., 2013; Morgan et al., 2011; MacCannell, 1976; Smith, 2009).

Recent Thai research links community tourism management to participation, cultural conservation, and collaborative networks ( Hoonpadoungrat et al., 2025). Research in Wang Krod also connects digital communication planning with community culture and the evaluation of promotional activities ( Chumnumwat & Chanvichai, 2024). Regenerative tourism extends sustainability debates toward the capacity of places and communities to flourish ( Bellato et al., 2023). Together, these perspectives frame post-pandemic recovery as more than renewed visitor growth.

In this study, authenticity concerns who controls cultural representation; CBT concerns participation and benefit distribution; governance concerns decision-making power; and marketing concerns whose narratives reach visitors. Borderland conditions connect these processes across ethnic and institutional boundaries. Participation may remain tokenistic when consultation provides no influence over decisions ( Arnstein, 1969), while cultural promotion may encourage commodification without community control ( Cohen, 1988). These concepts guide interpretation rather than constitute a tested causal model. Low-carbon transport, local procurement, and heritage protection are proposed planning priorities, not measured outcomes of this study.

## 3. Materials and methods

The qualitative and interpretive paradigm was used in this study to generate an in-depth understanding of the experiences of the stakeholders relating to the transformation of cultural tourism in the Karen hill tribe village in Sangkhlaburi District Kanchanaburi Province, Thailand ( Denzin & Lincoln, 2018). Considering the exploratory nature of the research questions and the need to take a nuanced look at social processes, cultural dynamics, and institutional relationships, which are not easily quantified, qualitative inquiry was found to be the most suitable type of study. The study was conducted from February to May 2024. Semi-structured interviews were conducted with 23 purposefully selected key informants comprising three groups: 12 government officials, six provincial officials, and five community leaders, using the categories reported in Table 1. A detailed description of the key informants is presented in Table 1. A purposive sampling method was used to include perspectives from governance and community levels. The interviews took between 60 and 90 minutes, with participants consenting to have the interview recorded.

**Table 1.** Profile of key informants.

Category	Affiliation/Group	n	Role in Tourism Development
Government Officials	Tourism Authority of Thailand (TAT)	12	Policy formulation, destination promotion, planning
Provincial Officials	Kanchanaburi Provincial Office of Tourism and Sports	6	Local coordination, infrastructure, event management
Community Leaders	Karen, Mon, Burmese ethnic communities	5	Cultural preservation, community participation
**Total**		**23**	

Interviews were supplemented by field observations of cultural sites and tourism attractions in Sangkhlaburi District and a review of national and provincial tourism plans, promotional materials, and CBT project reports. Cross-checking these data sources helped increase the comprehensiveness of the findings and made them credible ( Lincoln & Guba, 1985). Thematic analysis followed Braun and Clarke’s (2006) six phases: familiarization, initial coding, searching for themes, reviewing themes, defining and naming themes, and reporting. Data source triangulation, member checking, and reflexive journaling ( Denzin & Lincoln, 2018) were used to gain trustworthiness. Ethical approval was obtained before data collection (No. COE 66/011 – institutional ethics), and written informed consent was obtained from all participants. The research team, rather than participants, assigned ordinal theme scores reflecting the intensity and occurrence of themes in participant accounts, from 1 (very low) to 5 (very high). Means and standard deviations summarize these assigned scores descriptively; equal spacing between categories is not established, and the scores do not measure statistical significance or participant agreement. No inferential tests were conducted. The available methodological description does not specify coder numbers, independent ratings, or inter-rater agreement; scoring reproducibility therefore cannot be established.

## 4. Results and discussion

The findings show that the Karen Hill Tribe community (Sangkhlaburi) is experiencing a multi-faceted and complex transformation in tourism. The identified thematic dimensions are interrelated and include cultural and spiritual identity, challenges in governance, infrastructure limitations, challenges in marketing, and the potential of CBT. The descriptive statistics of each thematic dimension are detailed in Table 2 for all 23 respondents and are useful for understanding and adding depth to the qualitative data.

**Table 2.** Descriptive statistics for thematic dimension scores (n = 23).

Thematic Dimension	Mean	SD	Min	Max/Interpretation
**Cultural & Spiritual Identity**	4.17	0.78	3	5 ( Figure 2)
**Governance Issues**	3.57	1.20	2	5 ( Figure 2)
**Infrastructure Issues**	3.61	1.31	2	5 ( Figure 2)
**Marketing Limitations**	3.22	1.17	2	5 ( Figure 2)
**CBT Potential**	3.78	0.85	3	5 ( Figure 2)

The mean scores assigned to each thematic dimension are shown for the three stakeholder levels in Figure 1. Specifically, Cultural and Spiritual Identity had the highest mean score (M = 4.17, SD = 0.78) overall, indicating the prominence of cultural heritage as the most basic element of the destination to which the tourism appeal is connected. CBT Potential was rated second highest overall (M = 3.78, SD = 0.85), while Provincial Officials showed the greatest appreciation for CBT as a development pathway (M = 4.00). Infrastructure Issues (M = 3.61, SD = 1.31) and Governance Issues (M = 3.57, SD = 1.20) had similar mean scores, indicating perceived constraints on tourism development. Marketing Limitations had the weakest mean of all areas (M = 3.22, SD = 1.17), with observed variation between groups that was not statistically tested.

**Figure 1.**
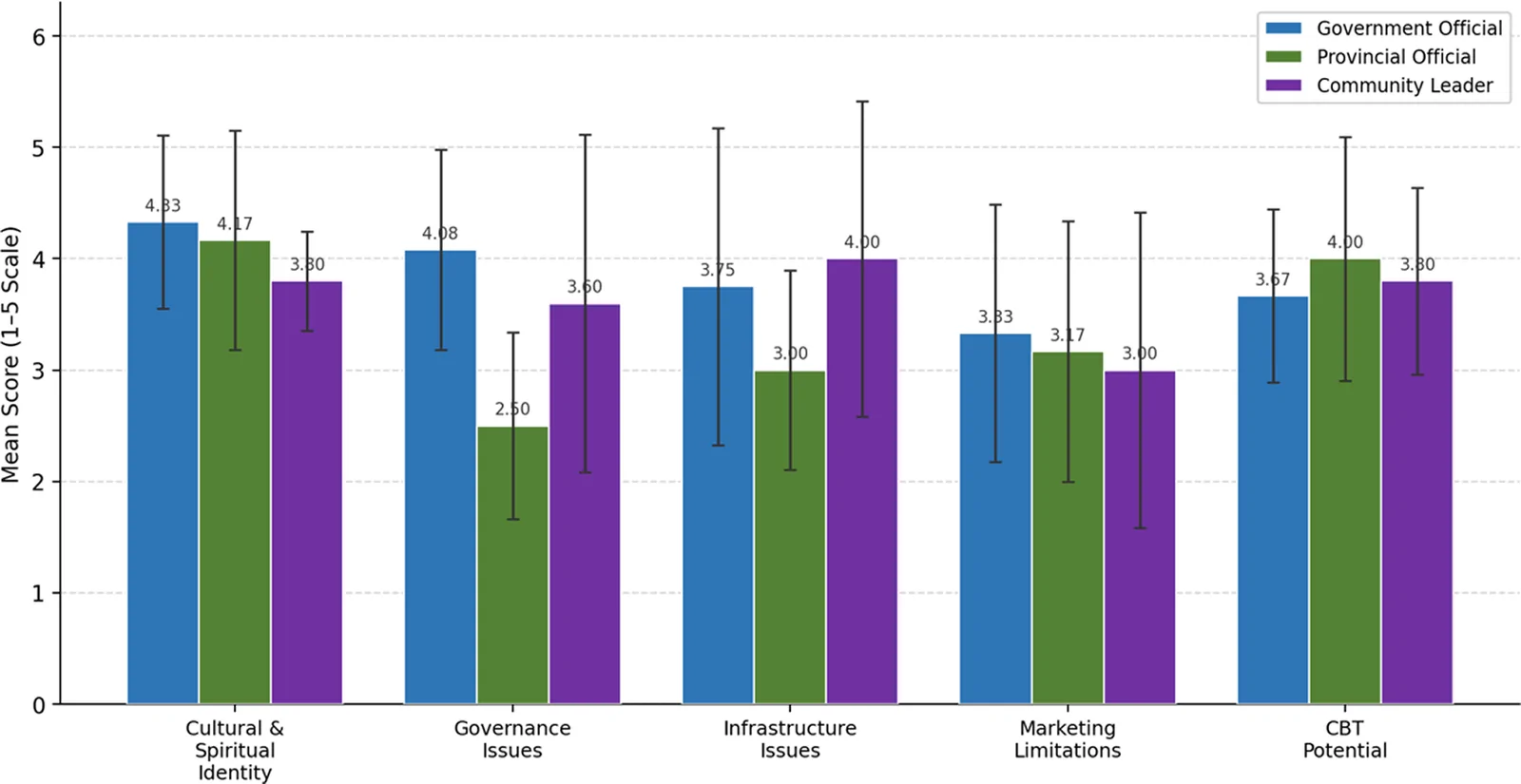
Mean theme scores by stakeholder category (error bars = ±1 SD; n = 23).

A horizontal bar chart of the overall mean scores across the five thematic dimensions is shown in Figure 2, the center of the scale shown as a reference line. The scores for all five themes exceeded the midpoint, indicating average researcher-assigned scores above 3; this does not establish majority agreement. It is noteworthy that Cultural and Spiritual Identity is the highest ranked in terms of ratings, as in qualitative results, it has been shown as the irreplaceable core of the tourist values of the destination, confirming what was previously found in many other dimensions of the destination.

**Figure 2.**
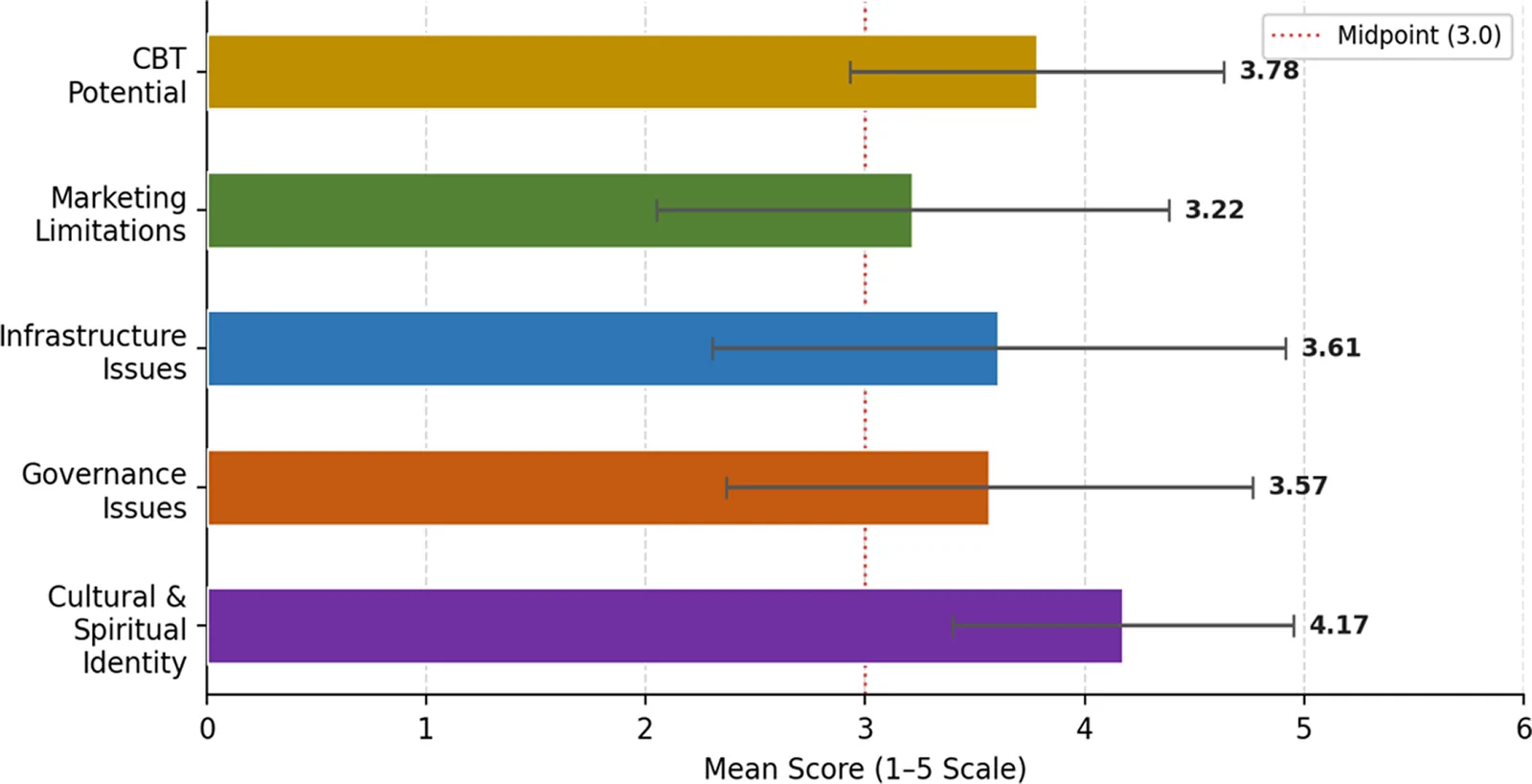
Overall mean scores across five thematic dimensions (n = 23; midpoint reference line at 3.0).

Figure 3 presents the distributions of researcher-assigned scores across the five themes. For Cultural Identity, 78% of participant accounts were assigned scores of 4 or 5, showing good convergence on this scale. The scores for the three issues (Governance, Infrastructure, and Marketing Limitations) are far more spread, suggesting varied stakeholder perspectives; institutional position and experience offer possible interpretations, rather than demonstrated explanations. The CBT development direction is also quite concentrated with high CBT Potentials (3–5), and here, there is also the consensus that it is a viable and good development direction, with no participant account assigned a score below 3.

**Figure 3.**
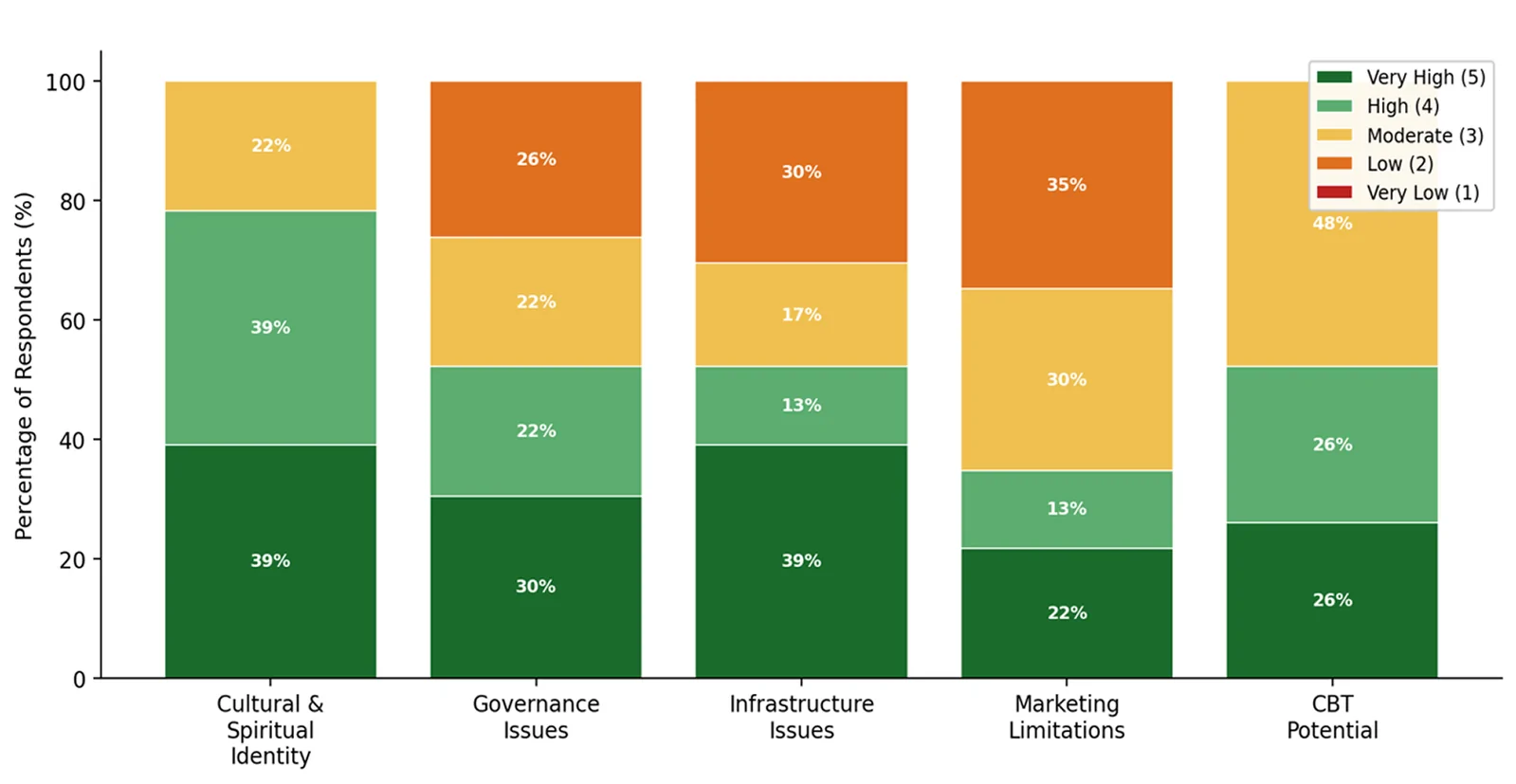
Score distribution by thematic dimension (% of respondents, n = 23).

A radar chart that juxtaposes the three stakeholder groups’ mean score profiles on all five dimensions is shown in Figure 4. Government Officials had the highest ratings for Governance Issues (M = 4.08) and were aware of the institutional problems of coordination and gaps in policy implementation. There is a low governance score (M = 2.50) for provincial officials, which may be due to a focus on operational aspects or satisfaction with the coordination mechanisms at the provincial level. The perceived lived experience of physical inaccessibility, as rated by Community Leaders, was the highest of all groups (M = 4.00). The differences in perceptions of governance between the national and provincial governments, as shown in the radar chart above, can be interpreted alongside Bramwell and Lane’s (2011) discussion of governance challenges, but the descriptive scores alone do not establish governance failure.

**Figure 4.**
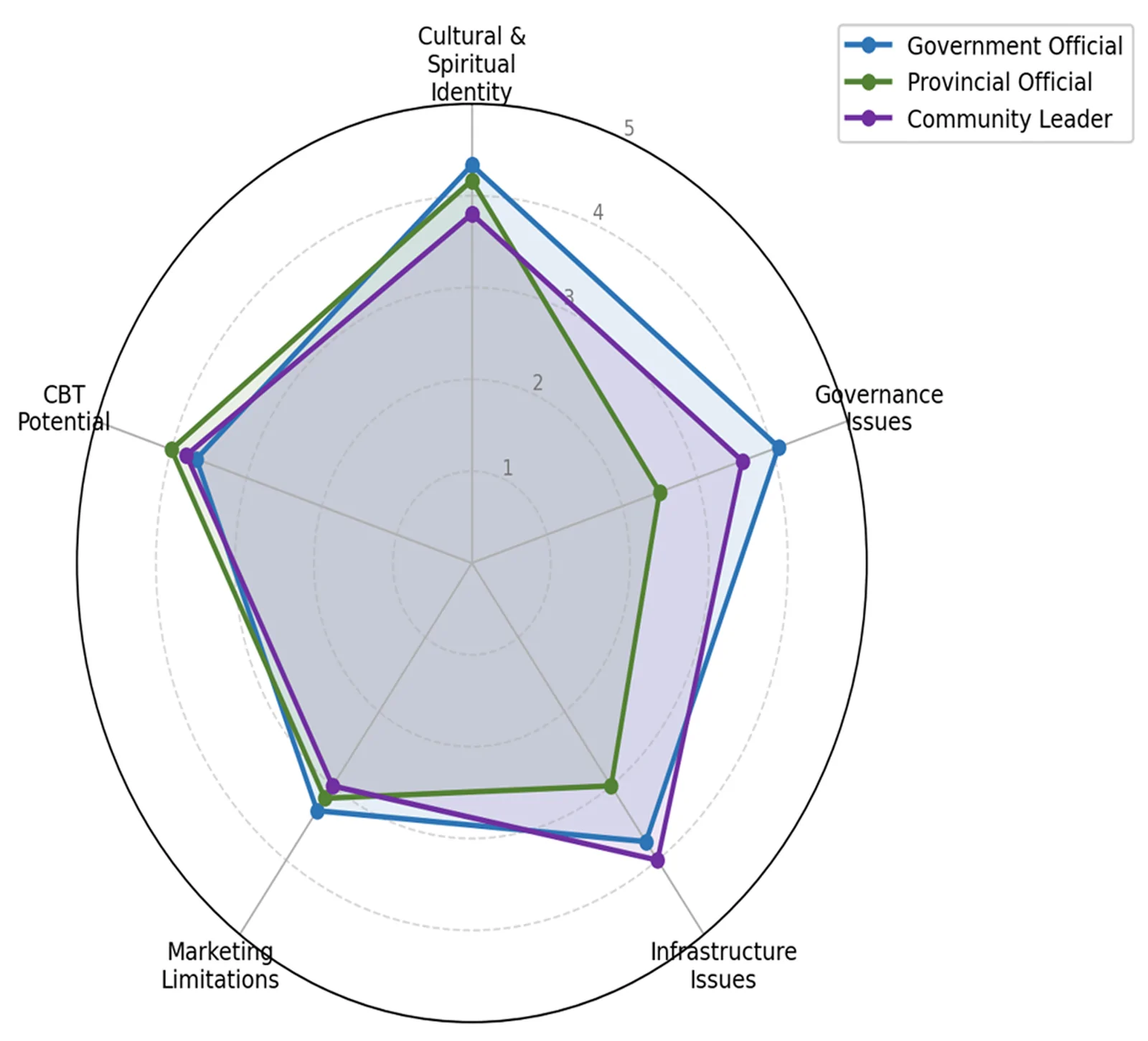
Radar profile of mean theme scores by stakeholder category.

The dot plot in Figure 5 shows researcher-assigned scores for individual participant accounts for each theme, colored by each stakeholder group, with horizontal lines representing group means. This plot depicts the variation within the group that the aggregate means obscured. Within the Government Official group, scores assigned to some TAT administrators’ accounts are still fairly varied, for example, 3 versus 5 in the Governance Issues case, which suggests varying institutional perspectives and impressions of the effectiveness of planning. In the case of Marketing Limitations, the overall scores range from 2 to 5 ( Table 2), indicating variation in the assessed prominence of marketing constraints.

**Figure 5.**
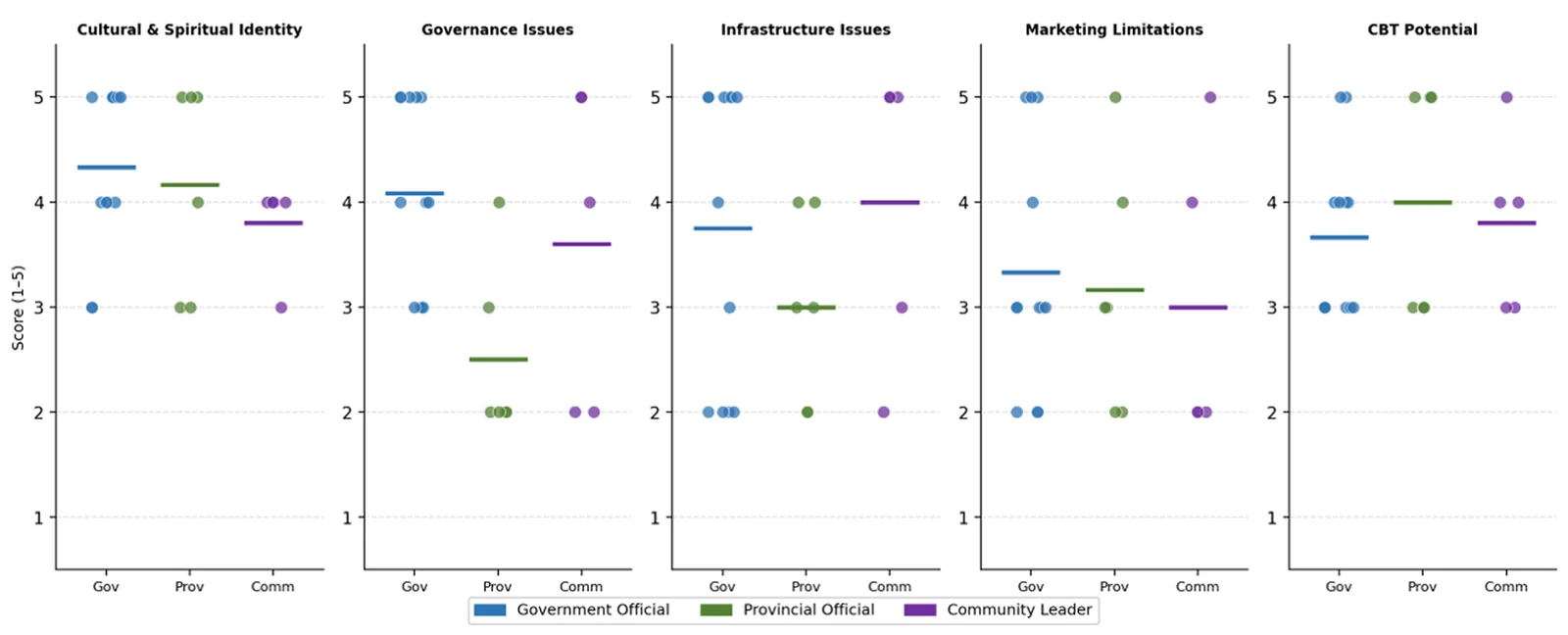
Individual respondent scores by theme and stakeholder category (horizontal lines = group means).

Table 3 presents a complete picture of the five thematic dimensions that combine the quantitative scoring pattern with the key qualitative issues identified by thematic analysis and selected quotations from the representatives. The figures and this table show that all five dimensions are recognized as important, with Cultural and Spiritual Identity having the highest overall mean and smallest SD, followed by CBT Potential in mean score; governance and infrastructure remain perceived structural constraints.

**Table 3.** Summary of thematic analysis findings.

Theme	Key Issues Identified	Illustrative Stakeholder Perspective
Cultural & Spiritual Identity	Heritage as tourism core; commodification risk; intergenerational knowledge	“Our traditions attract tourists, but their meaning must be preserved.” (Karen community leader)
Governance & Institutions	Top-down planning; coordination deficits; exclusion of community voices	“Policies exist, but local voices are often excluded.” (Provincial official)
Infrastructure & Accessibility	Poor road access; limited signage; inadequate visitor facilities	“Some places are very difficult for visitors to reach.” (TAT officer)
Destination Marketing	Over-focus on iconic sites; weak digital presence; external intermediary dependence	“Tourists know only a few places here.” (Local entrepreneur)
CBT & Sustainable Tourism	Strong social capital; community enthusiasm; need for institutional support	“We want tourism that benefits our community.” (Community elder)

The theoretical discussion relates these findings to the existing literature. The continued strong rating of Cultural and Spiritual Identity as the key dimension also aligns with Cohen’s (1988) emergent authenticity model and Richards’ (2011) notion of the need for communities to have interpretative agency over their heritage for cultural tourism to be sustainable. The stakeholder dimension in each of the three dimensions of governance perceptions diverges most strongly in the dimension of Governance Issues, which speaks to Arnstein (1969)‘s ladder of citizen participation’ and the perspective of governance coordination failures through the lens of Bramwell and Lane (2011). However, as noted earlier, the high CBT Potential scores across all groups are indicative of the work of both Murphy (1985) and Okazaki (2008), who developed frameworks for community-centered development efforts. Likewise, the within-group variability observed in Figure 5 suggests the contextual and positional nature of stakeholders’ governance perceptions identified by Jamal and Getz (1995). The infrastructure findings are similar to those of Sharpley (2002) and UNWTO (2019), while the marketing findings agree with those of Hays et al. (2013) and Morgan et al. (2011), who pointed to the importance of electronic presence as a competitive weakness of peripheral destinations.

A district forum could bring government agencies and community-selected Karen, Mon, and Burmese representatives into regular planning meetings, with published decisions and a mechanism for responding to community objections. Participating communities could agree transparent benefit-sharing rules, maintain a record of local procurement and household participation, and retain authority over the presentation of sacred practices. Digital marketing training should support community-managed content. Evaluation could track the proportion of community proposals adopted, local revenue retained, heritage complaints, visitor waste, and uptake of lower-carbon transport.

## 5. Limitations and scope for future research

This study had a few limitations. First, due to the qualitative interpretive design method used and the context of the setting (the Sangkhlaburi area), the results may not be statistically generalized to other locations within the borderland tourism region. The thematic scores presented in Figures 1– 5 and Tables 2– 3 are qualitative data derived from 23 purposively selected key informants on that scale and should be interpreted as a reflection of the intensity and frequency of themes and not as an established psychometric instrument. In applying this framework in other contexts, a contextual assessment should be undertaken to confirm the applicability of the identified dimensions. Second, several relevant stakeholder groups were not directly engaged, such as domestic and international tourists, private sector operators, and non-governmental organizations. Future studies, including survey studies that involve more respondents, would allow for more reliable inferential comparisons to be made based on these perspectives. Third, the cross-sectional design means that perceptions were observed at one time; scores would indeed benefit greatly from longitudinal studies to see how scores change over time as a result of governance changes or CBT interventions. Further research priorities include comparative research with other ethnic minority tourism destinations within the mainland Southeast Asian region to look for regional generalizability, further exploration of digital technologies and social media platforms to increase the sharing of community-led tourism experiences and measuring and evaluating participatory governance structures and how these influence the generation of measurable community-level benefits.

Community leaders accounted for only five of 23 participants (21.7%), while the government-official category accounted for 12 (52.2%). The findings therefore describe selected stakeholder perspectives and cannot represent the wider Karen community. The reported community category combines Karen, Mon, and Burmese representatives without an ethnic breakdown, preventing Karen-specific interpretation. Small, unequal groups and overlapping SD bars further limit group comparisons; SD bars show dispersion, not confidence intervals. The absence of documented coder agreement, a complete operational scoring rubric, and transcript-linked quotations limits the auditability of the qualitative scoring.

## 6. Conclusion

This study explored the changing phenomenon of cultural tourism in a Karen Hill Tribe village in the Sangkhlaburi District, Kanchanaburi Province, Thailand. Based on qualitative information derived from deepening insights through 23 key informant (KI) interviews and aspects of thematic scoring, this study sheds light on five interrelated dimensions that are important in the context of a culturally dispersed border area linked to tourism transformation: cultural and spiritual identity, institutional challenges of governance, infrastructure and accessibility issues, tourism marketing constraints, and tourism potential of the communities. The empirical findings that emerged throughout the five figures and the three tables point to the same perception of Cultural and Spiritual Identity as the main factor of tourism appeal (M = 4.17), followed by CBT Potential (M = 3.78), which was identified as a promising development pathway. Structural constraints are most marked in terms of Governance Issues (M = 3.57) and Infrastructure Issues (M = 3.61) and are revealed in the stakeholder group comparisons as key differences in perceptions of governance issues among government officials, provincial officials, and community leaders. This study emphasizes how cultural identity and spiritual heritage serve as commodities in the tourism industry and as dynamic and vibrant expressions of sustainable social and cultural living and intergenerational continuity. Sensitive, collaborative, and community-based tourism management, viewed as the engine of local cultural revitalization and economic empowerment, can be a partner in cultural change. The governance dimension reveals a perceived disconnect between national and provincial tourism policy and its local implementation, which requires the creation of participative planning platforms and multi-stakeholder committees, along with mechanisms for formalizing the community voice. This research contributes to the understanding of the significance of tourism development strategies that prioritize the conservation of culture, population welfare, and environmental sustainability in the backdrop of Thailand’s BCG Economy Model and its post-pandemic tourism recovery.

### Institutional review board statement

Ethical approval was obtained from the Ethics Committee in accordance with the Kasetsart University Research Ethics Committee (Approval Code: COE 66/011). The study received ethical clearance prior to data collection, and the authors acknowledge the governing bodies of Sangkhlaburi District, as well as all community members and local officials, for their valuable support and cooperation throughout the research.

## Data availability

The dataset supporting the conclusions of this article is available in the figshare repository. Dharawanij, N., & Yaiyong, A. (2026). The alternative cultural and CBT tour routing in Sangkhlaburi, Kanchanaburi, Thailand: Karen hill tribe village [Dataset]. figshare. https://doi.org/10.6084/m9.figshare.32125084.v1

This project contains the following file:

• Cultural and CBT - Raw Data.xlsx
• Cultural and CBT - Questionnaire.docx

Data are available under the terms of the Creative Commons Attribution 4.0 International license (CC-BY 4.0).
