# Supplementary material for: Stakeholder Perceptions of Cultural Tourism Transformation in a Karen Hill Tribe Village, Sangkhlaburi, Thailand
Source: F1000Res. 2026 Sep 16;15:1245. Originally published 2026 Jul 29. [Version 2] doi: 10.12688/f1000research.186965.2 (PMC13728442; doi:10.12688/f1000research.186965.2)
Supplement: Peer review history

# Reviewer report: Reviewer response for version 2

**DOI:** 10.5256/f1000research.210245.r521845

**Published:** 2026-09-25

**Authors:** Anas Pattaray, Endang Sri Wahyuni, Lalu Ratmaja

**Affiliations:** 1 Tour and Travel Business Management, Politeknik Pariwisata Lombok, Central Lombok Regency, West Nusa Tenggara, Indonesia; 2 Tour and Travel Business Management Study Program, Politeknik Pariwisata Lombok, Central Lombok Regency, West Nusa Tenggara, Indonesia

I have read the feedback from the authors. I have carefully reviewed the justifications and arguments regarding the corrections I provided. I believe this article is ready for indexing and can have a broader impact on the field of science. I have no further comments, as I believe the article is clear.

Is the work clearly and accurately presented and does it cite the current literature?

Partly

If applicable, is the statistical analysis and its interpretation appropriate?

Not applicable

Are all the source data underlying the results available to ensure full reproducibility?

Partly

Is the study design appropriate and is the work technically sound?

Partly

Are the conclusions drawn adequately supported by the results?

Partly

Are sufficient details of methods and analysis provided to allow replication by others?

Partly

Reviewer Expertise:

NA

We confirm that we have read this submission and believe that we have an appropriate level of expertise to confirm that it is of an acceptable scientific standard.

# Reviewer report: Reviewer response for version 2

**DOI:** 10.5256/f1000research.210245.r521844

**Published:** 2026-09-22

**Authors:** Warach Madhyamapurush

**Affiliations:** 1 Tourism and Hotel Management, School of Business and Communication Art, University of Phayao, Phayao, Thailand

Thank you to the authors for carefully revising the manuscript. Version 2 is clearer and more cautious than the original submission. The revised title is now more consistent with the scope of the study. The authors have also removed some unsupported statistical wording and clarified that the thematic scores were assigned by the research team rather than by the participants. The limitation concerning the unequal representation of the stakeholder groups is also clearly acknowledged.

 The practical implications are more specific and useful than in the previous version. The literature review has also been updated with more recent studies and now includes a brief conceptual synthesis. I also checked the reported means against the sample size (n = 23) and the subgroup sizes, and the figures appear to be consistent.

 However, several points raised in my previous review have not yet been fully addressed. There is also one new inconsistency that needs clarification before the findings and conclusions can be assessed with confidence.

**Major comments**

**1. The informant category has changed without explanation.**

 In Version 1, Table 1 identified the second group as “6 tourism entrepreneurs”. In Version 2, the same six informants are described as “Provincial officials” from the Kanchanaburi Provincial Office of Tourism and Sports. I could not find an explanation for this change in the Amendments section.

 There are also some related inconsistencies in the revised manuscript:

- Table 3 still identifies one quotation as coming from a “Local entrepreneur”, although this category no longer appears in Table 1.
- The Limitations section states that private sector operators were “not directly engaged”.
- The quotation attributed to a “Provincial official” in Table 3 (“local voices are often excluded”) is somewhat difficult to interpret alongside the fact that this group has the lowest governance score (mean = 2.50).

Please clarify who the six informants actually were and why their category was changed between the two versions. All quotations should also be attributed to stakeholder categories that are clearly identified in Table 1.

**2. Please clarify the composition of the six informants.**

 Please state clearly who these six people were, what their roles were, and why they are now classified as provincial officials rather than tourism entrepreneurs. This is important because the stakeholder composition affects how the results should be interpreted.

 Please also check all tables, quotations and the Limitations section to make sure that the stakeholder categories are consistent throughout the manuscript.

**3. The scoring procedure needs more detail.**

 The manuscript states that “the available methodological description does not specify coder numbers, independent ratings, or inter-rater agreement.” Since the authors have explained that the research team assigned the scores, more information should now be provided.

 Please clarify:

- How many researchers assigned the scores?
- Did they score the data independently?
- If there were differences in scoring, how were they discussed or resolved?
- What criteria or rubric were used to determine a score from 1 to 5?

The reader needs to understand how the qualitative interview material was converted into the numerical scores reported in the tables and figures.

**4. Please clarify the source of the scores.**

 If only one researcher assigned the scores, please state this directly.

 There is also an important point that needs clarification. The figshare dataset contains a file named “Questionnaire.docx”, while Figure 3 is labelled “% of respondents”. This raises the question of whether the scores were based on researcher coding of interview transcripts or on responses to a questionnaire completed by the participants.

 This distinction is important because it affects how the results in the tables and figures should be understood.

 I therefore recommend that the authors explain the scoring process in sufficient detail and include the complete scoring rubric as an appendix.

**5. Ethnic composition of the community informants**

 The manuscript states that the ethnic composition of the five community leaders is “unavailable”. Since the authors conducted the interviews themselves, I would expect this information to be available, unless there was a specific ethical reason for not recording or reporting it.

 Because the study is specifically framed around a Karen hill tribe village, please clarify how many of the community informants were Karen, and whether any were Mon or Burmese.

 If reporting the name of the village would create confidentiality concerns, the authors could instead provide a general description of the location. However, some basic information about the community studied would help readers understand the context of the research.

**6. More information is needed about the qualitative research process.**

 The qualitative methodology is still described rather briefly. Please provide more information about:

- the coding process and the unit of analysis;
- the language used during the interviews and, if applicable, the translation process;
- how the authors determined that sufficient data had been collected or that saturation had been reached; and
- whether member checking was conducted and, if so, how it was done.

These details are particularly important because the study uses a relatively small number of informants.

**7. The qualitative evidence should be expanded.**

 At present, the qualitative findings are supported by only five short and rather general quotations, with one quotation representing each theme. The quotations also do not have participant codes.

 I suggest adding several more anonymised quotations and identifying them with simple codes, for example, GO-03 or CL-02. It would also be useful to show whether participants from different stakeholder groups expressed similar or contrasting views.

 This would allow readers to see more clearly how the themes and interpretations were developed from the interview data.

**Minor comments**

- Please remove the remaining references to “consensus” in the Community-Based Tourism Potential paragraph and the phrase “good convergence”. The Conclusion also refers to “key differences in perceptions” between groups. Since the study does not appear to have formally tested differences between groups, I suggest describing these instead as differences or tendencies observed in the responses.
- Please check whether the cited references actually support the claims for which they are used. In particular:
  - King and Parnwell (2011), cited in relation to conditions specific to Sangkhlaburi;
  - Butler (1980), cited in relation to post-pandemic recovery;
  - Echtner and Ritchie (1993), which focuses on destination image measurement; and
  - Ministry of Tourism and Sports (2022), cited in relation to the Bio-Circular-Green Economy Model, while the reference listed appears to be a statistical annual report.
- Please also check the reference list:
  - Teo and Chee (2018) appears in the reference list but is not cited in the text.
  - The bibliographic details for Ashworth and Tunbridge (1990) and Lincoln and Guba (1985) appear to need correction.
  - Please check the year, volume and issue information for Gössling, Scott and Hall.
- The figshare dataset still uses the original title, “The Alternative Cultural and CBT Tour Routing…”. Please consider updating the dataset metadata so that the title is consistent with the revised manuscript.
- Low-carbon transport is proposed as one of the planning priorities. Please add at least one appropriate reference dealing with low-carbon tourism or low-carbon transport in tourism.
- Some sentences still need language editing. For example:
  - “The stakeholder dimension in each of the three dimensions of governance perceptions diverges most strongly in the dimension of Governance Issues.”
  - “However, as noted earlier” does not appear to connect clearly with the preceding discussion.
  - Please also correct the quotation marks around “Arnstein (1969)’s ladder of citizen participation”.
- In Table 2, the final column (“Max/Interpretation”) repeats “5 (Figure 2)” in every row. Please either rename this column as “Max” or provide an actual interpretation in this column.

**Overall assessment**

 The revised manuscript is clearly improved compared with the previous version. However, three issues still need particular attention:

1. the unexplained reclassification of the six informants;
2. the lack of sufficient detail about how the scores were assigned; and
3. the limited evidence describing the Karen community informants.

These issues are important because they affect how readers understand the sample, the scoring process, and the basis for the reported findings.

 Once these points have been clarified and the related inconsistencies have been corrected, I would be happy to reconsider the manuscript.

Is the work clearly and accurately presented and does it cite the current literature?

Partly

If applicable, is the statistical analysis and its interpretation appropriate?

Partly

Are all the source data underlying the results available to ensure full reproducibility?

Partly

Is the study design appropriate and is the work technically sound?

Partly

Are the conclusions drawn adequately supported by the results?

Partly

Are sufficient details of methods and analysis provided to allow replication by others?

Partly

Reviewer Expertise:

My research expertise is in tourism and hospitality management, with particular emphasis on community-based tourism and sustainable tourism development in Thailand. My research interests include cultural heritage tourism, community participation, tourism governance, regenerative and low-carbon tourism, and the assessment of the economic and social impacts of community tourism initiatives, including Social Return on Investment (SROI). This background enables me to critically evaluate the study’s conceptual framing, qualitative research design, methodological rigor, and the interpretation of community-based tourism issues.

I confirm that I have read this submission and believe that I have an appropriate level of expertise to confirm that it is of an acceptable scientific standard, however I have significant reservations, as outlined above.

# Reviewer report: Reviewer response for version 1

**DOI:** 10.5256/f1000research.206350.r506366

**Published:** 2026-08-08

**Authors:** Anas Pattaray, Endang Sri Wahyuni

**Affiliations:** 1 Tour and Travel Business Management, Politeknik Pariwisata Lombok, Central Lombok Regency, West Nusa Tenggara, Indonesia

1. The article raises a fairly specific context, namely the transformation of cultural tourism and *community-based tourism* (CBT) in the Karen community in the Sangkhlaburi border area, Thailand-Myanmar. The research yielded five main dimensions: cultural and spiritual identity, governance, infrastructure, marketing, and the potential of CBT. The article raises a fairly specific context, namely the transformation of cultural tourism and *community-based tourism* (CBT) in the Karen community in the Sangkhlaburi border area, Thailand-Myanmar. The research yielded five main dimensions: cultural and spiritual identity, governance, infrastructure, marketing, and the potential of CBT.

 The scientific novelty has not been explicitly explained. The authors have not clearly shown the differences in this study from previous studies on cultural tourism, CBT, and tourism governance in Thailand. In addition, the title emphasizes "alternative routes of cultural tours", but the results of the study do not present a route design, map, points of visit, distance, travel time, or route validation.

 Explicitly describe the gap in the article's research and new contributions. If the focus is on tourism transformation and CBT, the title needs to be adjusted. If you continue to use the concept of "alternative routes", the author needs to present the method of preparing the route, map, location selection criteria, and validation results.

 2. The article uses several relevant theories, such as border tourism, cultural authenticity, heritage tourism, CBT, collaborative governance, and community participation. The literature of Timothy, Cohen, Wang, Richards, Murphy, Okazaki, Arnstein, Bramwell, and Lane has been used as a theoretical basis.

 Literature reviews still tend to be a series of expert opinions and have not yet formed a critical synthesis. The relationship between the concepts of authenticity, CBT, governance, marketing, and border tourism has not been formulated in a clear conceptual framework. Up-to-date literature and empirical studies specifically addressing CBT, ethnic communities, power relations, and the distribution of tourism benefits are still limited. 

 Organize your literature reviews by theme, not by authors. Add up-to-date literature on power relations, participation tokenism, cultural commodification, CBT benefit distribution, tourism digitalization, and post-pandemic tourism resilience. Include a complete bibliography and make sure all citations are consistent.

 3. An interpretive qualitative approach is appropriate for understanding stakeholder experiences, cultural identities, community participation, and institutional relationships. Data collection was carried out through semi-structured interviews, observations, and document analysis. The author also mentions triangulation, member examination, reflective journals, and ethical approval.

 There is an inconsistency in the number of informants. The script states 23 informants, but the breakdown of the number of informant groups has the potential to number more than 23. Procedures for interviews, observations, and document analysis are also written repeatedly. The thematic analysis procedure has not explained the coding process, the formation of the theme, the language of the interview, the translation, the number of encoders, or the handling of interpretation differences. The use of ordinal scores of 1-5 for qualitative themes also does not have an operational definition, scoring procedure, or test of assessor consistency.

 Improve and clarify the number of informants in each category. Add an informant profile table. Explain the coding stages, units of analysis, theme formation process, data saturation, interview language, and the role of each researcher. If a score of 1–5 is maintained, include the score rubric, the scoring, the reliability procedure, and the reason for using the average and standard deviations.

 4. The results identified five relevant dimensions and presented average scores for cultural identity, CBT potential, infrastructure, governance, and marketing. Cultural and spiritual identity obtained the highest scores, while the potential of CBT became a development direction that was quite accepted by stakeholders. The discussion also sought to link results with the theory of authenticity, participation, and governance.

 Provide informant quotes to support each theme and show differences in perspectives between groups. Replace the term "significant" with "prominent" or "substantive" if no statistical testing is performed. Clearly separate empirical results, interpretations, and recommendations.

 5. Articles have mentioned implications for inclusive governance, community capacity building, participatory planning, digital marketing, comparative research, and longitudinal research. The author also acknowledges the limitations of generalizations, purposive samples, cross-sectional designs, and the lack of involvement of tourists, the private sector, and non-governmental organizations.

 Formulate practical operational implications, such as the establishment of multi-stakeholder forums, community representation mechanisms, benefit-sharing, cultural heritage protection, sustainability indicators, and evaluation systems. Also explain the theoretical contribution of the article, for example, the development of a CBT governance model in multiethnic border areas.

 The novelty is not yet clear, the title does not match the content, the number of informants is inconsistent, the theme scoring process is not transparent, the evidence of informant citations is limited, and the language and structure still need to be improved.

Is the work clearly and accurately presented and does it cite the current literature?

Partly

If applicable, is the statistical analysis and its interpretation appropriate?

Not applicable

Are all the source data underlying the results available to ensure full reproducibility?

Partly

Is the study design appropriate and is the work technically sound?

Partly

Are the conclusions drawn adequately supported by the results?

Partly

Are sufficient details of methods and analysis provided to allow replication by others?

Partly

Reviewer Expertise:

NA

We confirm that we have read this submission and believe that we have an appropriate level of expertise to confirm that it is of an acceptable scientific standard, however we have significant reservations, as outlined above.

# Reviewer report: Reviewer response for version 1

**DOI:** 10.5256/f1000research.206350.r506362

**Published:** 2026-08-03

**Authors:** Warach Madhyamapurush

**Affiliations:** 1 Tourism and Hotel Management, School of Business and Communication Art, University of Phayao, Phayao, Thailand

**1. Alignment between the title and the study scope**

 The current title suggests that the study develops or evaluates a tour-routing framework. However, the manuscript primarily reports stakeholder perceptions across five thematic dimensions without presenting route design, spatial mapping, or itinerary development. To improve consistency between the title and the actual content, the authors should either revise the title to better reflect the study focus (e.g., *Stakeholder Perceptions of Cultural Tourism Transformation in a Karen Hill Tribe Village, Sangkhlaburi, Thailand*) or incorporate a clearly defined tour-routing component that is explicitly derived from the findings.

**2. Consistency of participant numbers and institutional affiliations**

 The participant information requires clarification. The sample sizes reported in the Methods section do not correspond to the stated total number of participants. While Table 1 indicates 23 participants (12 government officials, 6 tourism entrepreneurs, and 5 community leaders), the narrative description appears to total 28 participants. In addition, the references to the “board of tourism” are not adequately explained, and the names of the participating government agencies differ between sections of the manuscript. These inconsistencies should be reconciled to ensure transparency and accuracy.

**3. Transparency of the qualitative scoring procedure**

 The manuscript would benefit from a more detailed explanation of how the thematic scores were generated. Since the scores appear to have been assigned by the research team rather than directly by participants, this should be stated explicitly wherever the scoring system is introduced. The authors should also report the number of coders involved, describe the coding process, and provide evidence of inter-rater reliability (e.g., percentage agreement or Cohen’s kappa). If only one researcher performed the scoring, this should be acknowledged as a methodological limitation. In addition, the manuscript should adopt one consistent interpretation scale throughout and include the complete scoring rubric as an appendix.

**4. Interpretation of descriptive findings**

 Some statements imply statistical inference that is not supported by the analytical approach. As no inferential statistical tests were conducted, conclusions referring to “significant differences” between stakeholder groups should be removed. Given the relatively small subgroup sizes (12, 6, and 5 participants) and the overlapping ±1 SD error bars shown in Figure 1, the results should be interpreted as descriptive patterns rather than statistically verified differences. Comparisons between groups should therefore be expressed more cautiously as observed tendencies or relative patterns.

**5. Representation of community stakeholders**

 The composition of the participant group should be discussed more explicitly as a study limitation. Community representatives account for only 5 of the 23 participants, whereas government officials represent more than half of the sample. Consequently, the findings should be interpreted as stakeholder perspectives rather than as representative of the broader Karen community. If possible, the authors should also specify how many of the community participants were Karen, as distinct from Mon or Burmese representatives.

**Minor Revisions**

- The observation procedures described in the Methods section contain repetitive text and should be streamlined. The apparent inconsistency between “participant observation” and “non-participant observation” should also be resolved.
- The terminology relating to community-based tourism should be standardised throughout the manuscript. The term **community-based tourism (CBT)** should be introduced at first mention and used consistently thereafter.
- The literature review would benefit from incorporating more recent scholarship (2022–2025), particularly studies addressing post-COVID community-based tourism, regenerative tourism, low-carbon tourism, and recent developments in Thai CBT research.
- The citation to *(Dharawanij & Yaiyong, 2026)* appears to refer to the authors’ own dataset rather than an independent scholarly source. This citation should be reconsidered or replaced with more appropriate supporting literature.
- The discussion of Murphy (1985) should be revised to more accurately reflect the original concept of a community-based development approach rather than characterising it as an externally driven growth paradigm.
- Several statements would benefit from clearer wording to avoid ambiguity or internal inconsistency. For example, expressions such as “serious issues but not a hindrance” should be reformulated, and the competing claims regarding the theme receiving the greatest consensus (Cultural Identity versus Community-Based Tourism) should be reconciled.
- Finally, a thorough English-language revision is recommended to improve readability by reducing redundancy, eliminating awkward phrasing, and ensuring consistency in academic style throughout the manuscript.

Is the work clearly and accurately presented and does it cite the current literature?

Partly

If applicable, is the statistical analysis and its interpretation appropriate?

Partly

Are all the source data underlying the results available to ensure full reproducibility?

Partly

Is the study design appropriate and is the work technically sound?

Partly

Are the conclusions drawn adequately supported by the results?

Partly

Are sufficient details of methods and analysis provided to allow replication by others?

Partly

Reviewer Expertise:

My research expertise is in tourism and hospitality management, with particular emphasis on community-based tourism and sustainable tourism development in Thailand. My research interests include cultural heritage tourism, community participation, tourism governance, regenerative and low-carbon tourism, and the assessment of the economic and social impacts of community tourism initiatives, including Social Return on Investment (SROI). This background enables me to critically evaluate the study’s conceptual framing, qualitative research design, methodological rigor, and the interpretation of community-based tourism issues.

I confirm that I have read this submission and believe that I have an appropriate level of expertise to confirm that it is of an acceptable scientific standard, however I have significant reservations, as outlined above.
